# A case of palatal adenoid cystic carcinoma with late recurrence

**DOI:** 10.3399/bjgpopen17X100785

**Published:** 2017-04-05

**Authors:** Dixa B Thakrar, Wai Sum Cho, Fiona Bishop

**Affiliations:** 1 Medical Student, University of Leicester Medical School, Centre for Medicine, Leicester, UK; 2 ENT Specialist Registrar, Department of Otolaryngology, Head and Neck Surgery, University Hospitals of Leicester, Leicester, UK; 3 GP, Market Harborough Medical Centre, Leicestershire, UK

**Keywords:** head and neck, cancer, recurrence

## Introduction

Adenoid cystic carcinoma (ACC) is a slow-growing, indolent cancer which normally develops in the head and neck salivary glands, but occasionally in the uterus.^1^ It can present with swelling, pain, and paraesthesia, as well as other symptoms such as epistaxis, nasal obstruction, depending on the location of the tumour.^2^ It accounts for 1% of the malignant tumours of the head and neck region.^3^ Classical management involves surgery and subsequent radiotherapy.^4^ The researchers describe a case of ACC of the minor salivary glands of the palate and nasopharynx that recurred 8 years following completion of treatment.

## Case report

A 57-year-old female presented to her GP with a 3-month history of left-sided catarrh and epistaxis from her left nostril. Clinical examination was unremarkable and the patient was initially diagnosed with sinusitis. However, the symptoms did not resolve following treatment for sinusitis. On further examination, her dentist noted left palatal swelling and referred her to the maxillofacial clinic by which time she had been suffering from these symptoms for 18 months. In hindsight, her epistaxis might have been a warning sign, and on reflection, the GP highlighted the need to take new epistaxis seriously. Clinical examination by the maxillofacial team revealed diffuse palatal swelling of the hard palate. Subsequent magnetic resonance imaging (MRI) showed a palatal tumour extending into the floor of the left nasal cavity and projecting into the left maxillary antrum through the medial wall. Biopsy of the palate showed an invasive tumour indicative of an ACC of minor salivary glands in the palate. Staging was T4N0M0.

She underwent a left hemimaxillectomy where the palate and floor of the nasal cavity were excised. The defect was covered with a removable obturator. She also had postoperative radiotherapy.

The patient remained in remission for 8 years. She underwent several surgeries during this time such as alar repositioning surgery to help reduce facial asymmetry and augmentation rhinoplasty to help support the nasal collapse that was secondary to the hemimaxillectomy and radiotherapy. She also had fat grafting to her upper lip to improve the lip seal. As a result of her disease process and treatment, she had Eustachian tube dysfunction and had a number of grommets inserted. She experienced problems in accessing an adequate palatal obturator requiring referral to Birmingham Dental School. Counselling from local hospice charity LOROS was also sought to help the patient come to terms with the psychological and physical impact of major and disfiguring surgery.

**Figure 1.**
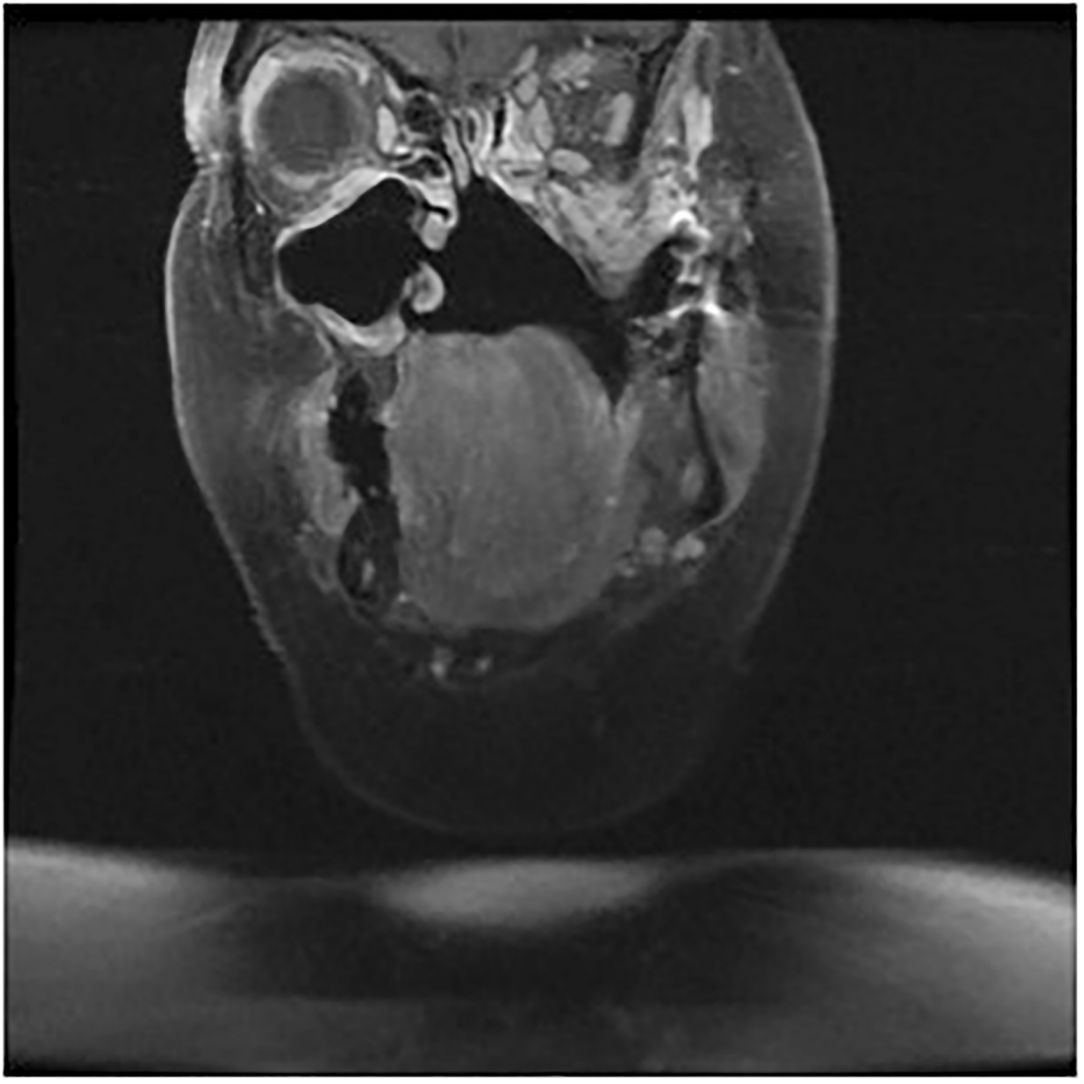
Coronal CT scan showing recurrence of tumour involving left masticator space.

After 8 years of being in remission, she presented to the GP with a tingling and burning sensation of her left mandible and tip of tongue. Clinical examination did not show any lesion in the oral cavity and oropharynx with no cervical lymphadenopathy. The patient was subsequently referred to the consultant maxillofacial surgeon who had a low index of suspicion for recurrence at this late stage so investigations were not urgently undertaken. The chest X-ray was organised by the GP at the request of the maxillofacial team and an MRI scan was organised by the maxillofacial team. Fortunately, the personal list system at the practice ensured that the GP was well informed and able to act promptly. However, even after MRI scanning, the diagnosis of recurrent disease was still unclear so a multidisciplinary team discussion regarding the need for biopsy took place. Ultimately, at biopsy she was found to have recurrent disease in the left masticator space extending up to the base of the skull (Figure 1). In addition, there was involvement of the left trigeminal nerve, explaining the patient’s unusual sensations. Computed tomography (CT) of the thorax additionally showed possible solitary metastasis, with a diameter of 1.5 cm in the left upper lobe, which was subpleural in location, though chest X-ray had been unremarkable.

**Figure 2.**
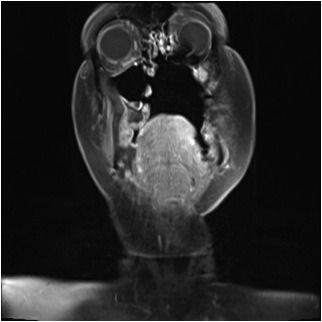
Coronal CT imaging following radical surgery to remove tumour.

Following a further multidisciplinary meeting, the patient underwent a left selective neck dissection, craniofacial resection including a lip split mandibulotomy, and reconstruction using a left radial forearm free-flap. Figure 2 shows the CT scan following the surgery.

Ten months after surgery, the patient underwent left video-assisted thoracoscopic surgery involving wedge resection of the subpleural, left upper lobe nodule. Histological examination confirmed this to be ACC. Since then, the patient has been in remission for 18 months. While medically she is in remission, she suffers from the psychosocial implications of the facial deformity following the surgery and the discomfort with the prosthesis. This continues to affect her quality of life, her confidence to be in public places, and ability to eat.

## Discussion

ACC is an uncommon neoplasm with an age-adjusted incidence rate of 4.5 cases per 1 000 000 person-years.^5^ Although ACC may represent a low burden on the health services relative to other cancers due to its lower prevalence, it considerably impacts the quality of life of the affected individuals and their families.

ACCs have been found to recur. Recurrence occurs in approximately 60% of patients of which 50% are clinically evident within 2 years of treatment.^6^ The authors would like to raise awareness that ACC has a tendency to cause perineural invasion as well. Hence, patients may present with neuropathic symptoms as seen in this case. Examination of cranial nerves is vital during assessment of these patients if recurrence is suspected.

In general, patients are followed for up to 5 years after completion of treatment to assess for any recurrence. This patient, however, presented with a recurrence 8 years postoperatively. This case can be used to raise awareness of patients presenting with recurrence many years down the line. This is especially relevant since some centres may not necessarily provide long-term follow-up.^7^ From the literature, follow-up for patients with ACC ranges from 4–14 years.^8^ GPs and consultant maxillofacial surgeons need to maintain a high index of suspicion for recurrence as ACC may recur outside the standard follow-up window. The tumour’s propensity for perineural invasion explained why this patient presented with neuropathic symptoms as a sign of recurrence. It is also important to note that the symptoms can be neuropathic, with no findings on clinical examination. Should there be any concerns about the possibility of recurrence, a referral to the head and neck surgical team is advised as MRI needs to be the investigation of choice and often such specialist views must be requested by ear, nose, and throat/maxillofacial surgeons.

The cosmetic and functional effects following radical maxillofacial surgery could have a profound impact on patients as well. It is important to be aware of the psychosocial implications of this and offer support to patients. As ACC is relatively rare compared to other cancers, there are no specific support groups related to this cancer. However, this patient did receive psychosocial support from LOROS, a Leicestershire charity, which delivered six sessions of one-to-one counselling. She found this very helpful and her only regret was not being offered more intensive counselling. Currently, she receives ongoing support from her GP who is well known to her. Such a disfiguring disease is rare and support from the community generally lacking. Referring such patients to cancer support groups locally and seeking more specific online support groups may be beneficial to the patient. While maintaining personal support may not be feasible in the long term, regular support groups with patients who are suffering with, or have suffered from head and neck cancers can be implemented within the community.

## Conclusion

ACC is a cancer with multiple presentations and can recur even many years down the line. Patient symptoms may simply be neuropathic in nature without evidence of any clinical lesion. It is always advised to always have a high index of suspicion and to seek surgical advice if there are any uncertainties.
